# Stress breaks universal aging behavior in a metallic glass

**DOI:** 10.1038/s41467-019-12892-1

**Published:** 2019-11-01

**Authors:** Amlan Das, Peter M. Derlet, Chaoyang Liu, Eric M. Dufresne, Robert Maaß

**Affiliations:** 1 Department of Materials Science and Engineering and Frederick Seitz Materials Research Laboratory, University of Illinois at Urbana-Champaign, Urbana, Illinois 61801 USA; 2 Condensed Matter Theory Group, Paul Scherrer Institute, Villigen, PSI 5232 Switzerland; 3 Advanced Photon Source, Argonne National Laboratory, Lemont, IL 60439 USA

**Keywords:** Glasses, Metals and alloys

## Abstract

Numerous disordered materials display a monotonous slowing down in their internal dynamics with age. In the case of metallic glasses, this general behavior across different temperatures and alloys has been used to establish an empirical universal superposition principle of time, waiting time, and temperature. Here we demonstrate that the application of a mechanical stress within the elastic regime breaks this universality. Using in-situ x-ray photon correlation spectroscopy (XPCS) experiments, we show that strong fluctuations between slow and fast structural dynamics exist, and that these generally exhibit larger relaxation times than in the unstressed case. On average, relaxation times increase with stress magnitude, and even preloading times of several days do not exhaust the structural dynamics under load. A model Lennard-Jones glass under shear deformation replicates many of the features revealed with XPCS, indicating that local and heterogeneous microplastic events can cause the strongly non-monotonous spectrum of relaxation times.

## Abstract (web summary)

Thermal annealing of metallic glasses is known to cause a universal increase of the relaxation time with sample age. Here, however, the authors show how a mechanical stress disrupts this universal response, leading to highly non-monotonous structural dynamics with time.

## Introduction

Significant structural evolution is a ubiquitous property of glassy materials, including polymeric glasses, oxide glasses, molecular glasses, and metallic glasses (MGs). The origin of this time-dependent evolution, typically referred to as aging, is the inherently out-of-equilibrium state, where local and thermally activated excitations reconfigure the atomic or molecular structure to lower the overall energy of the material^1^. These local relaxation processes lead to a time- and temperature-dependent change in the macroscopic properties, such as strength, ductility, toughness, elastic constants, volume, refractive index, stored excess enthalpy, or viscosity, and may involve significant property deterioration. Due to the complex atomic structure of glassy materials, quantification of aging mostly relies on assessing the aforementioned macroscopic parameters, or in selected cases, it is possible to probe dominant relaxation mechanisms in the glassy state via, for example, dielectric or mechanical spectroscopy^2–4^. While loss modes provide some understanding for how equilibration toward a lower energy state or toward the supercooled liquid may be facilitated, assessing the detailed picture of structural activity during aging in the glassy state remains an experimental challenge.

Recent progress in coherent scattering methods has addressed this shortcoming via X-ray photon correlation spectroscopy (XPCS) or electron correlation spectroscopy that allow the direct measurement of the structural dynamics at the interatomic or interparticle length scale in disordered systems^5,6^. Via the analysis of an intensity autocorrelation function, the structural relaxation time *τ* is obtained at any time. This approach has enabled deep insights into the structural dynamics of a number of systems and processes, including colloidal suspensions or gels^7,8^, ferro-fluids, ferromagnets and ferroelectrics^9–11^, polymeric glasses^12^, diffusion in crystalline alloys and phase transformations^13,14^, or aging in MGs^15–21^. Common to many of these studies is the use of a stretched exponential function that phenomenologically describes the relaxation behavior, via both the relaxation time *τ* and the stretching exponent *β*. One remarkable finding in the field of colloidal gels and MGs has been the observation that the dynamics of thermally activated relaxation processes follows a universal time-waiting time–temperature superposition^15,16,19,20,22^, which captures the increase in structural relaxation time with waiting time *t*_w_ via a model function that allows collapsing all data onto a single curve, independent of microscopic details of the system. For the MGs considered here, this means that the thermally activated atomic-scale relaxation dynamics is insensitive to the structural state, as macroscopically captured with density or microscopically described with the degree of medium and short-range ordering. Further support for such universal aging behavior has been provided by computer simulations that showed how *τ* increases linearly or sublinearly with *t*_w_^23^—a property also known for numerous polymeric glasses^24^ and spin glasses^25^, thereby underlining the wide applicability of the time-waiting time superposition across structural and nonstructural glassy systems.

**Fig. 1:**
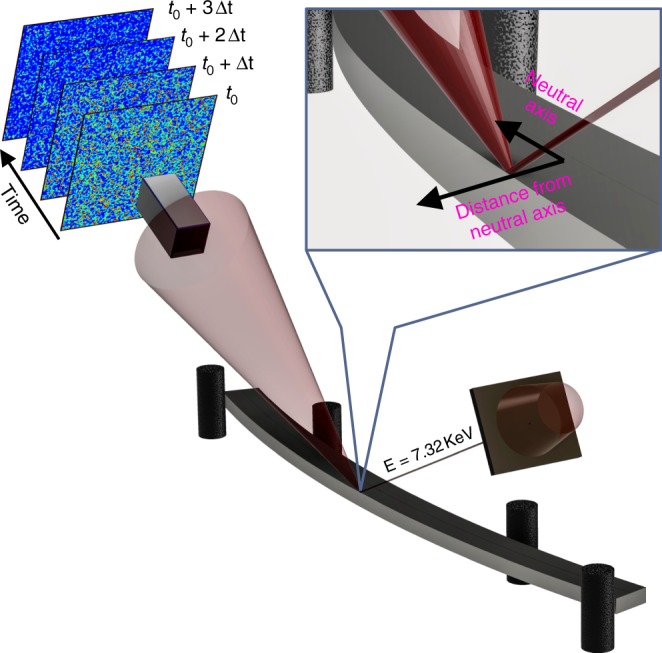
In situ four-point bending test. A coherent X-ray beam from a synchrotron source is focused and slitted down to form a 10-μm × 10-μm probe on the sample surface. The X-ray beam position away from the neutral axis determines the sign of the applied stress. Photons from only 1° of scattered angular range are collected for correlation spectroscopy

Here, we show how the application of stress breaks this universality in a Zr-based MG. We conduct an in situ XPCS four-point bending test, as schematically depicted in Fig. 1, to investigate the atomistic dynamics induced by a mechanical stress within the nominally elastic regime of the material. A partially coherent X-ray beam is used to probe the first peak of the structure factor and to reveal the time-dependent signature of atomic-scale activity in the MG at different stress magnitudes and signs on both sides of the neutral axis of the bending beam. The statistical correlation of the speckle patterns as a function of time and stress shows how the application of stress leads to relaxation times that generally are slower than those in the unstressed state. As a function of time, relaxation times vary strongly with time and can thus not be captured via the time-waiting time–temperature superposition. Furthermore, molecular dynamics (MD) simulations indicate that similar nontrivial fluctuations in speckle patterns can be caused by microplastic processes, suggesting that significant structural evolution is occurring even in the nominally elastic regime of the glass. More generally, these results show how the application of an external stress promotes fundamentally different structural dynamics than during unbiased thermal activation.

## Results

### Thermally activated and universal aging at room temperature and zero load

**Fig. 2:**
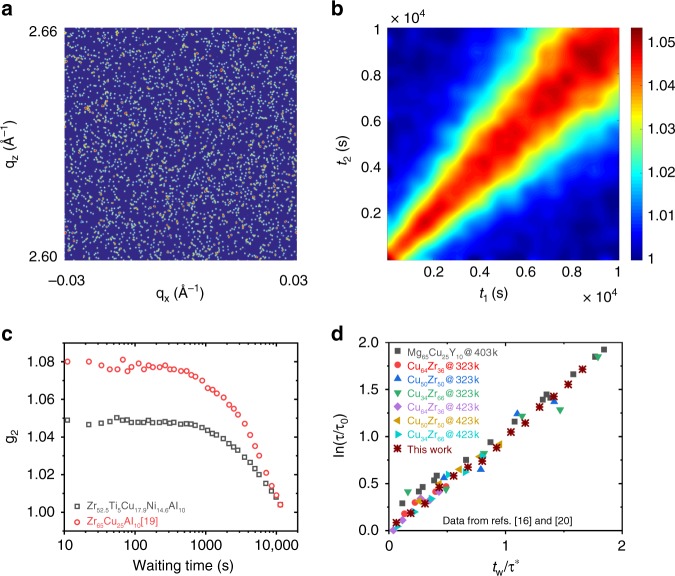
Intensity autocorrelation functions and universal scaling. **a** An example of an experimental speckle pattern, **b** a two-time correlation function for the here-studied alloy obtained at room temperature and zero stress, **c** one-time correlation functions for two different Zr-based MGs measured at room temperature and zero stress, and **d** a rescaling of experimental data from (**b**) and refs. ^16,20^, which demonstrates universal aging

Before turning our attention to the detailed stress-dependent dynamic behavior of the investigated MG, we briefly introduce some important aspects of the conducted experiments. In the following section, we exploit that changing scattered X-ray intensities over a very small range of the scattering vector, q, can be used to trace the structural evolution over an arbitrary variable, which here is time elapsed after loading the MG to a stress within the elastic regime. Thus, the comparison of structural activity at two different times can be done by comparing the corresponding speckle patterns, an example of which is displayed in Fig. 2a, with a two-time correlation function (TTCF). The TTCF between speckle patterns measured at times *t*_1_ and *t*_2_ is given by

1 $$C\left( {t_1,t_2} \right) = \frac{{\left\langle {I\left( {p,t_1} \right)I\left( {p,t_2} \right)} \right\rangle _{\mathrm{p}}}}{{\langle {I\left( {p,t_1} \right)_{\mathrm{p}}I\left( {p,t_2} \right)} \rangle _{\mathrm{p}}}}.$$

Here, 〈*I*(*p*)〉_p_ denotes the average of the pixel intensity *I*(*p*), over all pixels in the speckle pattern. Conventionally, XPCS measurements on MGs are performed during heating from room temperature to the glass-transition temperature *T*_g_, and further into the supercooled liquid regime^15–21^. In such a protocol, a complete decorrelation between the speckle patterns occurs within relatively short times (~10^2^–10^4^ s) when approaching *T*_g_, due to the increasing activation of *β*- and *α*-processes^26^. Under the condition where the width of the diagonal trace of the TTCF is monotonously and mildly increasing due to aging, or when aging is absent during stationary dynamics, a one-time correlation function or the intensity autocorrelation function, *g*_2_,

2 $$g_2\left( t \right) = \frac{{\left\langle {\left\langle {I\left( {p,t_{\mathrm{w}}} \right)I\left( {p,t_{\mathrm{w}} + t} \right)} \right\rangle _{t_{\mathrm{w}}}} \right\rangle _p}}{{\left\langle {\left\langle {I\left( {p,t_{\mathrm{w}}} \right)} \right\rangle _{t_{\mathrm{w}}}^2} \right\rangle _p}},$$

has been used extensively to characterize structural dynamics in both glasses, magnetic materials, and phase-change materials^27^. Here, *g*_2_(*t*) is the average of *C*(*t*_1_, *t*_2_) for *t*_1_ − *t*_2_ = *t*. The one-time correlation, *g*_2_(*t*), can be fitted by using the Kohlrausch–Williams–Watts (KWW) function of the form $g_2 = 1 + g_{2,{\mathrm{plat}}}{\rm{exp}}\left\{{-2\left( {\frac{t}{\tau }} \right)^\beta } \right\}$, where the contrast, *g*_2,plat_, is the product of an experimental factor and the square of the Debye–Waller factor^28^, and *τ* the relaxation time of the studied system (at *t* = *τ*, $g_2 - 1 = \frac{1}{{e^2}}g_{2,{\mathrm{plat}}}$). The shape parameter *β*, also called the stretching exponent, quantifies a deviation from pure exponential decay (*β* = 1), with *β* < 1 being characteristic of mass transport in liquids. The case of *β* > 1 is currently debated in the literature and may be related to the release of internal stresses^17^ or the dynamics of specific structural units^29^. In the following, we will focus on the application of Eq. (1), as it will become apparent that the averaging approach (Eq. (2)) and subsequent fitting to the KWW function may inappropriately quantify the thermally activated and stress-biased structural dynamics.

Figure 2b and 2c exemplify typical intensity autocorrelations in the form of *C*(*t*_1_,*t*_2_) for Zr_52.5_Ti_5_Cu_17.9_Ni_14.6_Al_10_ and in the form of *g*_2_(*t*) for Zr_52.5_Ti_5_Cu_17.9_Ni_14.6_Al_10_ and Zr_65_Cu_25_Al_10_^19^ measured at room temperature without any application of stress. The one-time intensity autocorrelation functions reveal a gradual decrease in the correlation with time, where a full decorrelation is obtained at *t* > 10^4^ s. These results represent the typically reported structural dynamics and its evolution with time, and have been shown for Zr-based^16,19–21^, Mg-based^15,16^, and Pd-based^17,18^ MGs. In general, the underlying TTCFs (as depicted in Fig. 2b) indicate a monotonously increasing relaxation time within the duration of the measurements, in that they quantify the aging behavior of the material. By using a time-waiting time superposition principle, it was proposed that the observed aging dynamics is universal for MGs^16^, which is manifested in a linear dependence on waiting time^19^ or an exponential relation with waiting time, irrespective of alloy composition or thermomechanical history of the alloys^16,19,20^. This can be demonstrated via the empirical scaling law *τ*(*T*, *t*_w_) = *τ*_0_(*T*)*exp*{*t*_w_/τ^*^}, where *τ*_0_ and τ^*^ are constants, and which allows collapsing all experimental data for thermally activated relaxation onto a single master plot. We reproduce this result in Fig. 2d with experimental data from ref. ^16^ and ref. ^20^. Figure 2d furthermore contains the data extracted from Fig. 2b, which were measured at room temperature and that excellently follows the empirical scaling law. As will be shown, stress breaks this universal picture of structural dynamics.

### Structural dynamics in response to stresses of different sign

In the following, we outline detailed results related to the structural dynamics in Zr_52.5_Ti_5_Cu_17.9_Ni_14.6_Al_10_ biased via the application of stresses within the Hookean regime at room temperature. This includes probing the MG under both compressive and tensile stresses. The effect of a preloading history and contrast fluctuations along the diagonals of *C*(*t*_1_, *t*_2_) are discussed in Supplementary Notes 1 and 2 of the Supplementary Information (SI) document. Since the experiments used a four-point bending geometry, we emphasize that the stress-sign variation is achieved by positioning the X-ray beam at different extremes (±2 mm) from the neutral axis (NA). Variations in stress magnitude are sampled via increasing the applied bending force on the sample, while always positioning the X-ray beam 50 μm away from the outer edge of the bent beam. This location at the edge of the specimen will be referred to as the outermost fiber. It is important to realize that the structural dynamics in MGs, as already apparent from Fig. 2, evolves with time, and it is thus practically impossible to trace the atomistic dynamics of the same volume element of the same specimen under two different stresses without a different structural history.

**Fig. 3:**
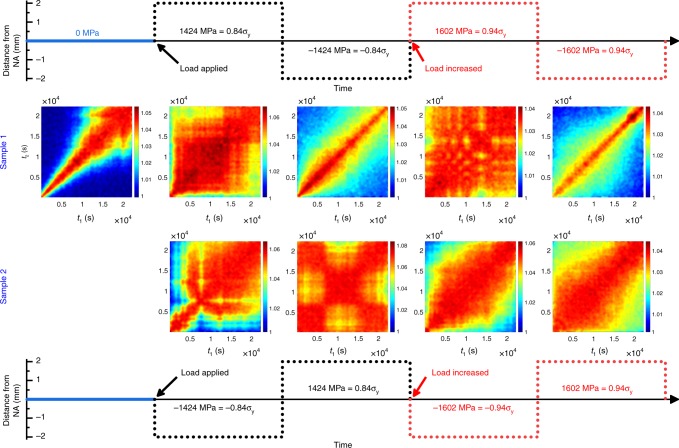
In situ bending with compressive and tensile stresses. Two-time correlation functions for two MG bars (Sample 1 and Sample 2), measured under different compressive and tensile stresses of different magnitude. The zero-load TTCF is also shown for Sample 1

A Zr_52.5_Ti_5_Cu_17.9_Ni_14.6_Al_10_ MG bending bar was loaded in two steps. Subsequent to speckle pattern measurements at *σ* = 0, a load was applied to bend the specimen, and the X-ray beam was positioned (by movement of the experimental stage) at the outermost fiber of the sample, probing a stress of 0.84*σ*_y_ or a tensile stress of 1424 MPa (*σ*_y_ = 1700 MPa^30^). After recording the speckle patterns at 0.84*σ*_y_, the X-ray probe was positioned onto the opposite extrema of the sample, and the speckle patterns at −0.84*σ*_y_ were thus monitored. Following these two measurements at ±0.84*σ*_y_, the sample was loaded further. This subsequent increase in the bending load corresponded to a stress of ±0.94*σ*_y_ in the outermost fibers. After this sequence of stresses for this sample (Sample 1), the inverse sequence but without the zero-stress condition was examined for a second sample (Sample 2) from the same alloy piece. The results are summarized in Fig. 3, where the TTCF plots are all scaled from 1 to 1 + *g*_2,plat_ to facilitate comparison. This choice of color scaling is motivated by our main interest to evaluate time-dependent reductions of the contrast, rather than changes in its absolute value.

The first sequence of stress states probed with Sample 1 displays a marked tension–compression asymmetry in the time-dependent structural activity. A tensile stress leads to virtually no resolvable structural dynamics within the experimental time window, and the average signal from the illuminated volume suggests structural constancy. This is different for the compressive stresses, for which initially some weak broadening (increase in relaxation times) is seen in the TTCF, followed by a narrowing (acceleration of the dynamics). Repeating the same experimental protocol in reversed order for Sample 2 clearly indicates that the apparent tension–compression asymmetry of Sample 1 is a feature of the particular experiment. Indeed, Sample 2 displays significantly broadened diagonal traces irrespective of stress sign and stress level, with some pronounced non-monotonic evolution of the time-dependent relaxation dynamics. The latter is occurring shortly after the application of the stress (0.84*σ*_y_). Based on Fig. 3, there is thus no observable correlation between stress sign and the induced structural dynamics at the here- covered timescales.

**Fig. 4:**
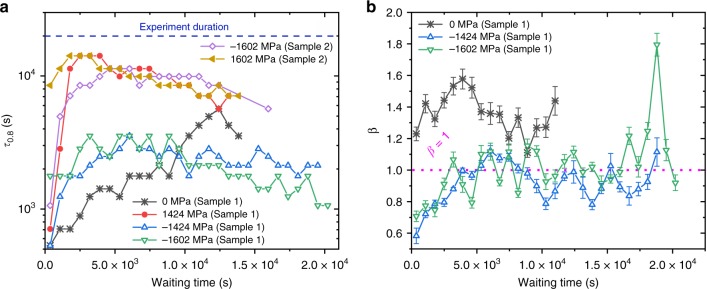
Time evolution of the relaxation times and shape parameter. **a** *τ*_0.8_ as a function of waiting time for different stress levels of the studied Zr-based glass. The duration of each XPCS experiment, effectively an upper bound to determining *τ*_0.8_, is also indicated. **b** The shape parameter *β* for momentary relaxation dynamics, with residual standard deviation errors as a function of waiting time for different stress levels. Tensile and compressive stresses are marked by solid and open symbols, respectively. The 0-MPa data are from Sample 1 in a completely unloaded condition

In addition to the TTCFs displayed in Fig. 3, a more quantitative representation is offered by the evaluation of momentary relaxation times as a function of waiting time. This can be done via studying the decorrelations along a perpendicular direction to the main diagonal^8,31^, or via the evaluation of decorrelations along horizontal traces, with only one time changing forward in time relative to the chosen waiting time at *C*(*t*_1_,*t*_1_). Due to the complex nature of the data, we chose here horizontal decorrelations starting at a given waiting time. Traditionally, in the KWW model, the relaxation time *τ* is defined as the time elapsed until the correlation (*g*_2_) has reached a numerical value of 1/*e*^2^ (~0.13) of its original value (*g*_2,plat_). This may be calculated by substituting *t* = *τ* in the KWW function. However, due to the slowed-down dynamics, this large reduction of contrast is often absent within the experimental window. To facilitate quantification, we choose to define the parameter *τ*_0.8_, which is the time it takes for the correlation function, *C*(*t*_1_, *t*_2_) to decay to 0.8 of its value on the main diagonal, *C*(*t*_1_, *t*_1_). We note that upon calculating momentary relaxation dynamics in the unloaded sample, *τ* and *τ*_0.8_ are found to be tightly correlated, thereby justifying *τ*_0.8_ as a semiempirical figure of merit. Figure 4 summarizes, whenever quantifiable, *τ*_0.8_ and *β* as a function of experimental time.

Figure 4a reinforces the conclusions made on the basis of the TTCFs in Fig. 3, namely that loading the material to the indicated stresses has a pronounced effect on slowing down the structural dynamics, in particular at short waiting times. The shape parameter, *β*, can only be reliably calculated for the unstressed sample and the compressive stress states of Sample 1. It is seen that *β* > 1 for the as-cast glass, whereas the application of a compressive stress reduces the shape parameter to values that initially are below 1 and at larger times fluctuate around 1. Thus, compressive loading leads to a change from a compressed decorrelation to a stretched decay, followed by simple exponential dynamics. While it is impossible to experimentally reveal the underlying physics for this change of *β*, recent computer simulations indicate that the value of *β* is linked to the predominant activity of differently sized structural units in the MG^29^. Indeed, ref. ^29^ demonstrates that compressed decorrelations are linked to the activity of clusters of icosahedra, whereas a stretched decorrelation is due to liquid-like isolated icosahedra. Thus, the applied stress may here disrupt parts of the icosahedral network, and the initial (*t*_w_ < 5 × 10^3^ s) time-dependent increase in *β* can be interpreted as a result of structural reconfigurations during the restoration of the icosahedral network.

Furthermore, the TTCFs shown in Fig. 3 (Sample 2) highlight a distinct time evolution of the correlation function, where decorrelation is followed by an evolution that regains values close to those originally seen at *C*(*t*_1_, *t*_1_). This was, within the precision of the experiments, not quantifiable for Sample 1. At −0.84*σ*_y_ (Fig. 3, Sample 2), a horizontal time trace starting at *t*_1_, *t*_2_ = 4000 s evidences first a correlation reduction from 1.057 to 1.02, which subsequently increases back to 1.053. Similar, but not as large, changes in the correlation are seen for the tensile stress of 0.84*σ*_y_ probed afterward. These variations in correlation occur at *t*_2_ − *t*_1_ ≫ 0 and are thus not defined by *g*_2,plat_, and a sensitivity to changes in thermal vibrations (mean-square displacement). Instead, the decrease and subsequent increase in *C*(*t*_*i*_, *t*_*j*_) along a horizontal or vertical trace in the TTCF signifies that the structure is regaining a statistically similar configuration.

### Structural dynamics in response to tensile stresses of different magnitude

**Fig. 5:**
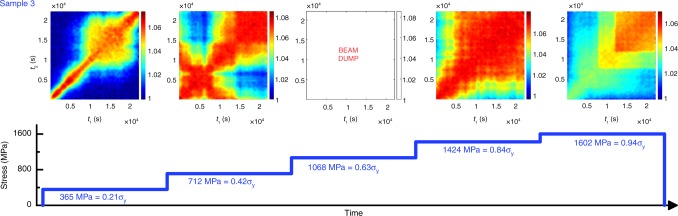
In situ bending with increasing stress. Two-time correlation functions for Sample 3 as a function of increasing tensile stress. The X-ray beam was lost at 1068 MPa

In addition to tracing the time-dependent evolution of the speckle patterns at different stress signs and magnitude, the stepwise increase of the tensile load in the outermost fiber of the bending beam is now considered. The probed stress levels are 356 MPa (0.21*σ*_y_), 712 MPa (0.42*σ*_y_), 1068 MPa (0.63*σ*_y_), 1424 MPa (0.84*σ*_y_), and 1602 MPa (0.94*σ*_y_). Figure 5 summarizes the obtained TTCFs for a stepwise load increase. The synchrotron beam was lost due to a machine error at 0.63*σ*_y_.

Overall, Fig. 5 shows similar features as highlighted in Figs. 3 and 4, with the addition of more extreme temporal fluctuations in the momentary relaxation dynamics at the two lower stress levels. The intermittent dynamics under stress is well exemplified with the data obtained at 0.21*σ*_y_ (Fig. 5). At 0.21*σ*_y_, the dynamics are fast enough to permit the accurate calculation of the relaxation time, *τ*. In this case, the TTCF begins with a region of fast dynamics (*τ*_0.8_ = 8 × 10^2^ s, *τ* = 3 × 10^3^ s) that slows down to *τ*_0.8_ = 5 × 10^3^ s or *τ* = 1.4 × 10^5^ s for ~10^4^ s and then speeds back up to *τ*_0.8_ = 2 × 10^3^ s or *τ* = 5 × 10^3^ s toward the end. Figure 5 also reveals abrupt variations in the contrast at the highest stress. Such intermittent contrast variations have been seen during martensitic phase transformations in crystalline alloys^14^, local but collective rearrangements in 2D nanoparticle monolayers^32^, as well as in MGs during cooling^17^, and are referred to as structural avalanches. Seen at the highest applied stress, the intermittent dynamics may thus indicate that the system is close to global yielding, i.e., shear banding^33,34^.

**Fig. 6:**
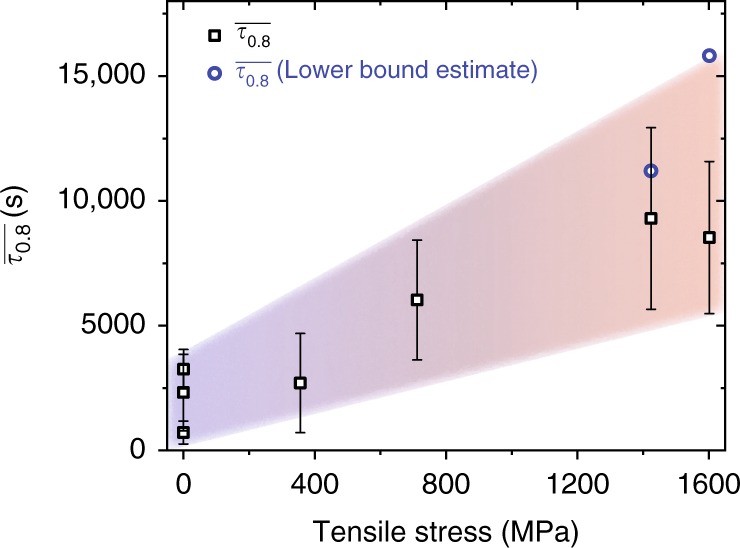
Stress dependence of the average momentary relaxation time. The average momentary relaxation time increases with applied tensile stress. Square symbols show standard deviations. Round symbols are lower-bound estimates based on the measurement duration of the corresponding TTCF

For all tensile-stress states, *τ*_0.8_ is calculated whenever possible and averaged. We observe a systematic increase in average momentary relaxation time $\overline {\tau _{0.8}}$ with increasing applied stress, which is summarized in Fig. 6 for all TTCFs that were recorded under tensile loading. Given the large fluctuations in dynamics within the experimental window, $\overline {\tau _{0.8}}$ has an expected significant standard deviation of 50%, as shown for square symbols. Figure 6 also contains two lower-bound estimates of $\overline {\tau _{0.8}}$. These values are from two experiments, where no significant decorrelation was observed during the experimental time. Thus, the experimental time serves as a lower estimate for $\overline {\tau _{0.8}}$, since any possible decorrelation in the future would always yield a higher value of $\overline {\tau _{0.8}}$. The aggregated data points strongly suggest on-average slower dynamics with increasing tensile stress from 0 to 0.94*σ*_y_. It should be noted that a regime of stationary dynamics (constant $\overline {\tau _{0.8}}$ at any stress) may be emerging at the long-time limit, which would mean that all stress-biased thermally activated dynamics has been exhausted. Verification of this long-time limit is, however, outside any experimental feasibility.

Beyond the results summarized in Fig. 6, the measured data do not reveal systematic dependencies, and even several days of preloading does not seem to exhaust the intermittent structural activity, as shown in the SI. This means that the information contained in the TTCF is a snapshot of a continuously changing dynamics within the sample. We note that this statement has to be seen relative to the experimental time of the XPCS measurements.

## Discussion

During thermal annealing of MGs, relaxation times have empirically been demonstrated to fall onto a single master curve with appropriate rescaling (Fig. 2d)^15,16,19,20^. In fact, similar findings have been reported for soft materials^9,22,35^, hinting toward a universal increase in the momentary relaxation times with sample age. Our results show that this property is not seen when a mechanical stress within the macroscopically elastic regime is applied.

The variation of the loading protocol and loading history pursued here, demonstrates a nontrivial sequence of relaxation spectra contained in successive TTCFs. During a given experimental window, the probed structure may lack any signature of activity, whereas a subsequent measurement, under the same loading conditions, can again reveal momentary relaxation times that are observable within the timescale of the experiment. As such, stress seems to stimulate slower structural activity that is described by short- and long-term fluctuations in the time-dependent decorrelations. A related analysis between different TTCFs, by using higher-order correlation functions (not shown here), reveals that the most pronounced correlation times are of the order of the experimental window of 6 h. This indicates that even larger experimental times would be required to capture the full range of decorrelations induced via stress.

Paying closer attention to the evolution of the TTCFs as a function of stress (Fig. 6), the data show how momentary relaxation times are short at low stresses, and temporarily even shorter than for the specimen without any loading history. This suggests that processes characterized by fast relaxation times may be quickly exhausted after the application of stress. Thus, the slow dynamics predominantly revealed in the experiments are only reflecting the experimentally accessible long-time portion of the entire relaxation-time spectrum activated by the stress bias. As such, the slow dynamics due to stress at long waiting times is not in contradiction with expected stress-accelerated structural activity for glassy systems^19,36^.

With the main conclusion being that a mechanical stress within the elastic regime breaks the universally scalable and monotonous thermally activated relaxation, we pose the question what microscopic mechanisms may be underlying the intermittent dynamics in the TTCFs. Since the sample is loaded at low temperatures (*T*/*T*_g_ = 0.44), and since it does not exhibit any macroscopic plastic flow (absence of shear bands, and no resolvable permanent bending), the structural activity must be caused by a large number of localized excitations that are of *β* type^37^. In view of the large illuminated volume element (10 × 10 × 25 μm^3^), the XPCS method is thus proposed to reveal the net dynamics of microplastic^38^ structural excitations that are heterogeneous in both space and time.

One way to test this proposition is to investigate the effect of both elastic deformation and plastic deformation on calculated speckle patterns obtained from an atomistic simulation. To this end, we use MD simulations of a model glass system, which naturally implies that any data represented in a TTCF would be restricted to orders of magnitude shorter timescales than in the experiments. However, atomistic simulations do give detailed information on the microscopic processes underlying high strain-rate athermal plastic processes^39,40^. More specifically, we use atomistic simulations to test the extremes of structural dynamics and its effect on a TTCF: elastic behavior is expected to yield close-to-static TTCFs, whereas plastic flow is expected to cause strain-dependent fluctuations in a TTCF.

With this idea in mind, we calculate a corresponding two-strain correlation function (TSCF) to reveal significant decorrelations with respect to strain—which at fixed strain rate is equivalent to time. The SI outlines in more detail how the TSCF is obtained. Albeit at substantially shorter timescales, this calculation allows verifying what potential microstructural processes may lead to a similar intermittent decorrelation as seen in the experiments.

**Fig. 7:**
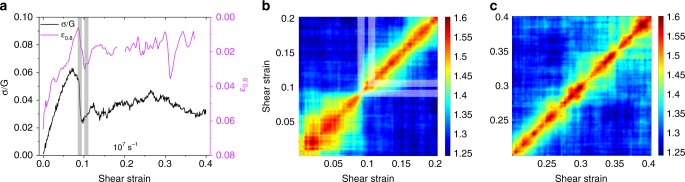
Atomistic simulations reveal the origin of intermittent dynamics. **a** Normalized shear stress along with *ε*_0.8_ momentary relaxation strains as a function of shear strain for a binary model glass strained at 10^7^ s^−1^ under simple shear. Note the inverted axis on the relaxation strain. **b** A two-strain correlation function from 0 to 0.2 in shear strain shows the transition from elastic to plastic flow at a shear strain of ca. 0.074, followed by fluctuations in relaxation strain with plastic flow. **c** Shear strains from 0.2 to 0.4 show fluctuating relaxation strains, decorrelation–recorrelation, as well as variation in contrast along the main diagonal. Similar results are found for a strain rate of 10^5^ s^−1^, which are displayed in the SI

We thus perform exceptionally long-time (μs) MD on a model binary Lenard–Jones glass. We investigate a considerably relaxed glass system, by applying a constant simple shear rate, and measure the shear-stress response. The details of the MD simulations are outlined in the “Methods” section, and additional results are shown in Supplementary Note 3. Figure 7a plots the shear stress and the momentary relaxation strain, *ε*_0.8_, as a function of shear strain. The simulations reveal an initially elastic stress–strain response followed by a transition to plastic flow with the typical varying flow regime. This behavior correlates well with the momentary relaxation strain and the TSCF in Fig. 7b, c. During elastic loading, large relaxation strains are seen in the TSCF that clearly reduce at the onset of significant plastic flow at a shear strain of around 0.1. At this point, *ε*_0.8_ finds its smallest value. We note that *ε*_0.8_ offers a peek into the very immediate future of the underlying atomic structure. This explains why ε_0.8_ may slightly precede the corresponding fluctuation in shear stress, and why it gradually decays toward a plastic instability.

The TSCF in Fig. 7b focuses on the initial 0–0.2 strain regime and Fig. 7c on the final strain of 0.2–0.4. Figure 7b, c shows strong fluctuations in the TSCF, increasing in magnitude at the later stages of deformation. A narrowing of the diagonal in the TSCF indicates strong periods of structural activity, which in this case underlie the predominantly athermal plasticity seen in the stress–strain response. This gives a clear indication for the underlying structural activity in the experimental TTCFs.

**Fig. 8:**
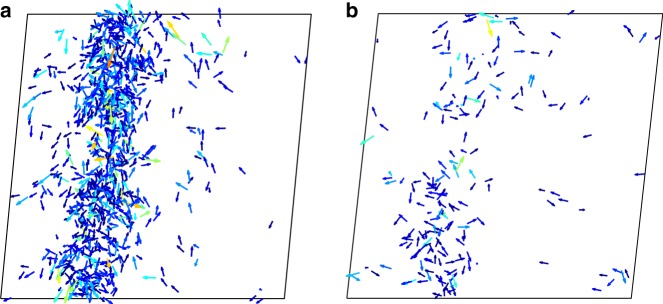
Localization of microplastic activity. Displacement maps comparing the atomic displacements caused by a 0.01 increment in strain at a strain rate of 10^7^ s^−1^. **a**, **b** Fast and slow dynamics, respectively. The corresponding strain regions are highlighted in the stress–strain graphs in this figure. Similar results are found for a strain rate of 10^5^ s^−1^, which are displayed in the SI

A detailed analysis of the atomic-scale structure and its link to the non-monotonous TSCF reveals information on the spatial distribution of structural process in the simulations. The highlighted two-strain regimes in Fig. 7a are now considered, where the first one corresponds to a large drop in stress and the second highlight corresponds to a gradual increase in stress (elasticity offset by constant plastic activity). The highlighted strain regimes are also indicated in Fig. 7b and correspond to a narrowing and broadening of the TSCF, giving a direct link between plastic activity and fluctuations in the TSCF. Detailed inspection of the atomic-scale activity reveals that the drops in stress (and pinching of the TCSFs) correspond to system-spanning localized structural evolution, whereas the plastic activity seen during the local stress rises (and the broadening of the TCSFs) corresponds to dilute delocalized structural evolution. Figure 8 exemplifies this by displacement maps that show atomic displacements due to an increase in shear strain by 0.01, from the two different highlighted segments of the stress–strain curve. The left panel shows the atomic-scale activity underlying the stress drop, whereas the right panel shows what occurs during the stress rise. It is apparent that the underlying large and localized plastic activity gives rise to the observed faster dynamics, i.e., the pinching of the TSCF and thus a smaller relaxation strain (*ε*_0.8_). On the contrary, smaller magnitudes of atomic displacements (Fig. 8b) that are less correlated and delocalized are the cause for the relatively slower dynamics. Similar changes of the TSCF are summarized for a lower shear strain rate in the SI. We note that the experiments probe a significantly larger volume than that investigated via simulations. The atomistic effects seen here are thus expected to be much more pronounced in the experiments.

We conclude that in situ X-ray photon correlation spectroscopy experiments reveal how the application of a stress within the elastic regime of a MG leads to non-monotonous structural dynamics. The strongly time-dependent evolution of the relaxation times does not follow the generally observed time-waiting time–temperature superposition known for many glassy systems that undergo aging due to thermally activated structural activity. Combining the experimental results with atomistic simulations conveys a picture in which a succession of both space and time heterogeneous microplastic activity is the origin of the strongly non-monotonous correlation functions. These results reveal for the first time the atomic-scale dynamics that must underlie the numerously reported structural changes seen after macroscopically purely elastic loading of MGs^41–47^. Given the distinctly monotonously evolving relaxation times due to thermally driven structural dynamics in many glassy systems, we anticipate that our results on MGs are generally valid for other structural glasses.

Future experimental efforts need to focus on bridging between the here-revealed timescales and actual structural changes, as well as establishing a comparative ground between structural dynamics revealed with XPCS and other standard methods, such as mechanical spectroscopy. While our findings introduce XPCS as a powerful tool to trace structural activity in response to stress-biased thermal activation, i.e., micro- or macro-plasticity, the results also urge for groundbreaking theoretical work that can describe the nontrivial stress-driven structural dynamics, and provide a formalism that connects structural processes to the time-dependent dynamics shown here.

## Methods

### Sample preparation

Vitreloy 105 (Zr_52.5_Ti_5_Cu_17.9_Ni_14.6_Al_10_, *T*_g_ of 402 °C at 20 K min^−1^ ^48^) was purchased in the form of 1.5-mm-thick plates from Liquidmetal^®^ and cut into bars of dimensions 87 × 4 × 1.5 mm^3^. The plates were ground flat to ensure dimensional tolerance of 2%. The as-cast surface was retained for examination with the X-ray probe. The amorphous nature of the samples was verified by using wide-angle X-ray diffraction in a Phillips XPert 2 diffractometer.

### Mechanical loading

A customized four-point bender was used to apply load on the bars prepared. A four-point bending geometry corresponds to the same bending moment and stress levels along the length of the bar, between the two internal pivots, allowing for large tolerance in the position of the X-ray beam along the length of bar. Bending also allows us to probe tension and compression stress signs within the sample bar without changing applied load. The stress in the material was controlled by changing the deflection at the pivots (see Fig. 1). The bender was loaded according to planned experimental conditions and observed under an optical microscope, to ensure stability of time of the equipment. Drift measured was on the order of 1 μm over 24 h, which was determined to not have an impact on the experiment.

### X-ray measurements

X-ray photon correlation spectroscopy measurements were carried out at the 8-ID-E beamline of the Advanced Photon Source at Argonne National Laboratory. A photon beam energy of 7.32 keV was used in reflection geometry with a scattering angle 2*θ* = 41.566°, corresponding to q = 2.63 Å^−1^. The partially coherent beam from an undulator source was formed into a probe of 3.5 × 10 μm^2^. A coherent beam of 150 μm in vertical size was focused to 3.5 μm by using a Beryllium compound refractive lens (ten parabolic lenses, with a radius of curvature of 0.2 mm) placed 1.65 m before the sample. At the scattering geometry used, this corresponds to a footprint of 10.23 × 10 μm^2^. A partially coherent flux of 2 × 10^9^ photons per second was obtained. The sample edges were identified by using knife-edge scans, and the beam was placed 50 μm away from the edge during the XPCS measurements. Speckle patterns are recorded on a 2D charge-coupled device detector of size 18 × 19 mm^2^, located 1 m from the sample. This corresponds to a Δ*θ* of 1° or a Δ*q* of 0.0622 Å^−1^. Approximately, 2000 speckle patterns were recorded per scan, each with an exposure time of 10 s and a write time of 1 s, corresponding to ~6 h in total elapsed time. The probed *q* range did not reveal any *q* dependence of the shown results.

### Analysis

The speckle patterns are analyzed by code developed at sector 8ID at the APS, to produce intensity autocorrelation functions as well as two-time correlation functions.

### MD simulations

A 32,000-atom 50:50 model Lennard–Jones (LJ) binary glass cube-shaped sample is used in the present simulations. The glassy structure is generated by an initial NVT quench from a well-equilibrated liquid down to a temperature just below the transition to an amorphous solid (within the MD timescale). The sample is then held at this temperature (0.95*T*_g_) for ~2 µs, resulting in significant structural relaxation, giving a final icosahedral content of the smaller atom species equal to 30%. The sample is then quenched to 0 K. This NVT preparation procedure is given in more detail in previous works^49,50^. Now, by using an NPT ensemble, the temperature is raised to 0.7*T*_g_, details of which can be found in past work^51^. At this temperature, a pure shear deformation is applied within the NPT ensemble at fixed strain rates spanning six orders of magnitude. This work considers two strain rates, which for a real material would correspond to a rate of ~10^5^ and 10^7^ s^−1^. The spatial coordinates of the evolving system at fixed strain intervals, are used to calculate a speckle pattern via a standard Fourier transform for reciprocal vectors within the shear plane. Because the material is a model LJ system, a constant atomic form factor is used for all atoms. This entire procedure results in a reciprocal-space structural ring, all of which are used to calculate the required two-time correlation functions. To remove the effect of the changing elastic state of the system (which would deform the ring), only the non-affine deformation component of the atomic coordinates is used, thus focusing only on the contribution of the athermal plastic events. More details can be found in Supplementary Methods.

## Supplementary information

Supplementary Information

## Data Availability

The raw data generated and analyzed as a part of this study are available from the corresponding author upon request.

## References

[1] Struik L. C. E. Doctoral thesis: physical aging in amorphous polymers and other materials. *Technische Hogeschool Delft* (1977).

[2] Qiao JC, Pelletier JM (2014). Dynamic mechanical relaxation in bulk metallic glasses: a review. J. Mater. Sci. Technol..

[3] Gotze W, Sjogren L (1992). Relaxation processes in supercooled liquids. Rep. Prog. Phys..

[4] Küchemann S, Maaß R (2017). Gamma relaxation in bulk metallic glasses. Scr. Mater..

[5] Sutton M (1991). Observation of speckle by diffraction with coherent x-rays. Nature.

[6] He L, Zhang P, Besser MF, Kramer MJ, Voyles PM (2015). Electron correlation microscopy: a new technique for studying local atom dynamics applied to a supercooled liquid. Microsc Microanal..

[7] Schosseler F, Kaloun S, Skouri M, Munch JP (2006). Diagram of the aging dynamics in laponite suspensions at low ionic strength. Phys. Rev. E.

[8] Fluerasu A, Moussaid A, Madsen A, Schofield A (2007). Slow dynamics and aging in colloidal gels studied by x-ray photon correlation spectroscopy. Phys. Rev. E.

[9] Robert A, Wandersman E, Dubois E, Dupuis V, Perzynski R (2006). Glassy dynamics and aging in a dense ferrofluid. Europhys. Lett..

[10] Shpyrko OG (2007). Direct measurement of antiferromagnetic domain fluctuations. Nature.

[11] Gorfman S (2018). Ferroelectric domain wall dynamics characterized with X-ray photon correlation spectroscopy. Proc. Natl Acad. Sci. USA.

[12] Orsi D (2010). Slow dynamics in an azopolymer molecular layer studied by x-ray photon correlation spectroscopy. Phys. Rev. E.

[13] Leitner M, Sepiol B, Stadler LM, Pfau B, Vogl G (2009). Atomic diffusion studied with coherent X-rays. Nat. Mater..

[14] Muller L (2011). Slow aging dynamics and avalanches in a gold-cadmium alloy investigated by x-ray photon correlation spectroscopy. Phys. Rev. Lett..

[15] Ruta B (2012). Atomic-scale relaxation dynamics and aging in a metallic glass probed by x-ray photon correlation spectroscopy. Phys. Rev. Lett..

[16] Ruta B, Baldi G, Monaco G, Chushkin Y (2013). Compressed correlation functions and fast aging dynamics in metallic glasses. J. Chem. Phys..

[17] Evenson Z (2015). X-ray photon correlation spectroscopy reveals intermittent aging dynamics in a metallic glass. Phys. Rev. Lett..

[18] Giordano VM, Ruta B (2016). Unveiling the structural arrangements responsible for the atomic dynamics in metallic glasses during physical aging. Nat. Comm..

[19] Küchemann S, Liu C, Dufresne EM, Shin J, Maaß R (2018). Shear banding leads to accelerated aging dynamics in a metallic glass. Phys. Rev. B.

[20] Wong K (2018). Universal aging characteristics of macroscopically and microscopically dissimilar metallic glasses. Acta Mater..

[21] Luttich M (2018). Anti-aging in ultrastable metallic glasses. Phys. Rev. Lett..

[22] Cipelletti L, Manley S, Ball RC, Weitz DA (2000). Universal aging features in the restructuring of fractal colloidal gels. Phys. Rev. Lett..

[23] Kob W, Barrat J-L (1997). Aging effects in a Lennard-Jones glass. Phys. Rev. Lett..

[24] Alegría A, Goitiandía L, Tellería I, Colmenero J (1997). α-Relaxation in the glass-transition range of amorphous polymers. 2. Influence of physical aging on the dielectric relaxation. Macromolecules.

[25] Rieger H (1993). Nonequilibrium dynamics and aging in the three-dimensional Ising spin-glass model. J. Phys. A.

[26] Zhao ZF, Wen P, Shek CH, Wang WH (2007). Measurements of slowβ-relaxations in metallic glasses and supercooled liquids. Phys. Rev. B.

[27] Chushkin Y, Caronna C, Madsen A (2012). A novel event correlation scheme for X-ray photon correlation spectroscopy. J. Appl. Crystallogr..

[28] Ruta B, Pineda E, Evenson Z (2017). Relaxation processes and physical aging in metallic glasses. J. Phys. Condens Matter.

[29] Wu ZW, Kob W, Wang WH, Xu L (2018). Stretched and compressed exponentials in the relaxation dynamics of a metallic glass-forming melt. Nat. Commun..

[30] Morrison ML (2007). Four-point-bending-fatigue behavior of the Zr-based Vitreloy 105 bulk metallic glass. Mater. Sci. Eng. A.

[31] Madsen A, Leheny RL, Guo H, Sprung M, Czakkel O (2010). Beyond simple exponential correlation functions and equilibrium dynamics in x-ray photon correlation spectroscopy. New J. Phys..

[32] Boucheron LS (2018). Stress relaxation in quasi-two-dimensional self-assembled nanoparticle monolayers. Phys. Rev. E.

[33] Maaß R, Klaumünzer D, Löffler JF (2011). Propagation dynamics of individual shear bands during inhomogeneous flow in a Zr-based bulk metallic glass. Acta Mater..

[34] Maaß R, Löffler JF (2015). Shear-band dynamics in metallic glasses. Adv. Funct. Mater..

[35] El Masri D, Berthier L, Cipelletti L (2010). Subdiffusion and intermittent dynamic fluctuations in the aging regime of concentrated hard spheres. Phys. Rev. E.

[36] Chen, K. & Schweizer, K. S. Stress-enhanced mobility and dynamic yielding in polymer glasses.* Europhys. Lett. ***79**, 26006 (2007).

[37] Yu H-B, Wang W-H, Samwer K (2013). The β relaxation in metallic glasses: an overview. Mater. Today.

[38] Maaß R, Derlet PM (2018). Micro-plasticity and recent insights from intermittent and small-scale plasticity. Acta Mater..

[39] Swayamjyoti S, Loffler JF, Derlet PM (2014). Local structural excitations in model glasses. Phys. Rev. B.

[40] Falk ML, Langer JS (1998). Dynamics of viscoplastic deformation in amorphous solids. Phys. Rev. E.

[41] Harmon JS, Demetriou MD, Johnson WL, Samwer K (2007). Anelastic to plastic transition in metallic glass-forming liquids. Phys. Rev. Lett..

[42] Park K-W (2008). Elastostatically induced structural disordering in amorphous alloys. Acta Mater..

[43] Packard CE, Witmer LM, Schuh CA (2008). Hardening of a metallic glass during cyclic loading in the elastic range. Appl Phys. Lett..

[44] Tong Y (2015). Structural rejuvenation in bulk metallic glasses. Acta Mater..

[45] Greer AL, Sun YH (2016). Stored energy in metallic glasses due to strains within the elastic limit. Philos. Mag..

[46] Sun YH, Concustell A, Greer AL (2016). Thermomechanical processing of metallic glasses: extending the range of the glassy state. Nat. Rev. Mater..

[47] Ross P (2017). Linking macroscopic rejuvenation to nano-elastic fluctuations in a metallic glass. Acta Mater..

[48] Glade SC (2000). Thermodynamics of Cu47Ti34Zr11Ni8, Zr52.5Cu17.9Ni14.6Al10Ti5 and Zr57Cu15.4Ni12.6Al10Nb5 bulk metallic glass forming alloys. J. Appl. Phys..

[49] Derlet PM, Maaß R (2018). Thermally-activated stress relaxation in a model amorphous solid and the formation of a system-spanning shear event. Acta Mater..

[50] Derlet PM, Maaß R (2017). Thermal processing and enthalpy storage of a binary amorphous solid: a molecular dynamics study. J. Mater. Res.

[51] Derlet PM, Maaß R (2018). Local volume as a robust structural measure and its connection to icosahedral content in a model binary amorphous system. Materialia.

