# Supplementary material for: Stress breaks universal aging behavior in a metallic glass
Source: Nat Commun. 2019 Nov 1;10:5006. doi: 10.1038/s41467-019-12892-1 (PMC6825140; doi:10.1038/s41467-019-12892-1)
Supplement: Supplementary file 1 — Supplementary Information [file 41467_2019_12892_MOESM1_ESM.pdf]

*Stress breaks universal aging behavior in a metallic glass*

Das et al.

# *Stress breaks universal aging behavior in a metallic glass*

Amlan Das[1], Peter M. Derlet[2], Chaoyang Liu[1], Eric M. Dufresne[3], Robert Maaß[1]*

1. Department of Materials Science and Engineering, University of Illinois at Urbana-Champaign, Urbana, Illinois 61801, USA.
2. Condensed Matter Theory Group, Paul Scherrer Institute, 5232 Villigen PSI, Switzerland.
3. Advanced Photon Source, Argonne National Laboratory, Lemont, Illinois 60439, USA.

*Corresponding author (rmaass@illinois.edu)

**Supplementary Information**

This supporting online material presents additional data from both experiments and atomistic simulations. Experimental data for the effect of long-time pre-loading of the studied Zr-based alloy is summarized in Supplementary Note 1. Supplementary Note 2 discusses fluctuations of the contrast along the main diagonal of the TTCFs. Additional data for a shear-deformation simulation at $10^5\ s^{-1}$ can be found in Supplementary Note 3. Details on the MD simulations and how the two-strain correlation function was constructed are found in Supplementary Methods.

## Supplementary Note 1

To further elucidate the strongly fluctuating momentary relaxation times at very long time scales, we display in Supplementary Figure 1, data that was obtained after a bar (Sample 4) had been loaded in the four-point bending rig for 6 days prior to the first XPCS measurement. Thus, the first TTCF obtained when probing the bent bar at the outermost fiber, equivalent to $0.84\sigma_\mathrm{y}$, represents the structural activity at $t > 144$ hours. Despite this long preloading time, relaxation times smaller than the experimental time-scale have not been exhausted. Moving the x-ray probe to the opposite side of the neutral axis ($-0.84\sigma_\mathrm{y}$) yields a signal that practically indicates a stationary structure, but an immediate repeat of the same measurement clearly shows that this only was a temporary phase in the life-span of the stressed glass, since the second TTCF obtained at $-0.84\sigma_\mathrm{y}$ again reveals some structural evolution.

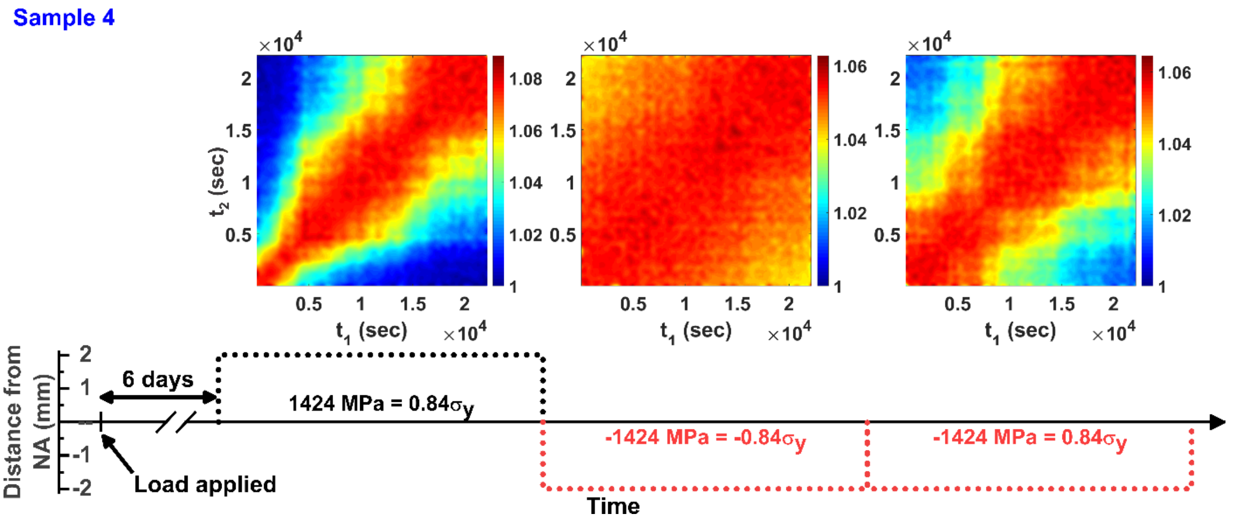


*Supplementary Figure 1:* **In-situ bending after long-time pre-loading.** Two-time correlation-functions for Sample 4 that was preloaded for 6 days prior to the XPCS measurements. Two subsequent measurements were conducted under identical compressive stress conditions.

## Supplementary Note 2

An additional property contained in Figures 3, 5 and Supplementary Figure 1 that we will address is the variation of the main diagonal trace, which would be the value of $g_{2,plat}$ for any locally extracted (momentary) decorrelation. Technically, the trace $t_1 = t_2$ is a self-correlation of the data and yields very high contrast values. This is circumvented in the analysis by creating an average value of the surrounding time entries with $t_1 = t_2 \pm t_a$, where $t_a$ is the acquisition time per speckle pattern. This value of momentary $g_{2,plat}$ can be interpreted through the Siegert relationship as a product of the non-ergodicity factor and an experimental contrast factor[1]. Under the conditions that the experimental settings remain unchanged, the contrast at the main diagonal is thus a measure of the mean-square displacement of the atoms in the illuminated volume element, occurring within the acquisition time (11 s). In the context of XPCS experiments conducted during heating, this reflects the gradual decrease of the peak-height of the first peak of the structure factor while the mean-atomic distance gradually smears out due to increased thermal stimulus[2]. Here the temperature does not change, and any changes in the momentary contrast value must thus originate from changes in the intermediate structure factor, linked to average interatomic distance variations. Such an assessment requires first a quantification of an experimental noise floor, which was determined with an $Fe_3Al$ single crystalline reference sample. Supplementary Figure 2 shows the normalized contrast fluctuations (NCF) as a function of time, which we define as:

$$NCF(t_1) = \frac{C(t_1,t_1) - C(t_1^{'},t_1)}{C(t_1^{'},t_1)} \qquad \text{(Supplementary Equation 1)}$$

It can be deduced from Supplementary Figure 2 that the NCF of both the unstressed and stressed MG are significantly stronger than in the $Fe_3Al$ reference crystal. This is justified by the fact that

the reference material is in a crystalline state and thus appears ‘frozen’ when compared to the metastable metallic glass. Further, there appears to be no significant difference produced in the contrast fluctuations, by the application of stress. Thus, we conclude that the short time-scale (less than acquisition time) fluctuations in the metallic glass, which dictate the value of the contrast, $C(t_1, t_1)$, are dominated by thermally-activated structural activity that is not affected by the applied stress. This is motivated by recent atomistic simulations that have shown how structural activity during a stress relaxation experiment proceeds statistically identical to purely thermally driven relaxation dynamics[3]. In other words, structural processes at short time scales are not strongly affected by the level of stress. However, while stress may not bias the structural excitations at very short time scales, it is clear from the above displayed non-monotonically evolving TTCFs that stress indeed affects the dynamic time-scales at much longer times than represented by $C(t_1, t_1)$ $(11\ s)$.

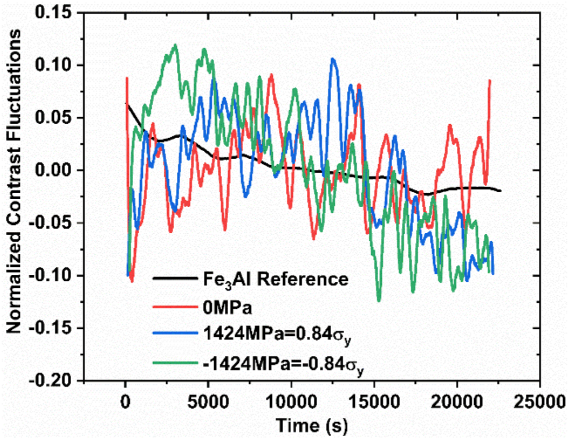


*Supplementary Figure 2:* **Contrast fluctuations of a crystal reference and the MG** Contrast fluctuations, subtracted from and subsequently normalized to their mean value are plotted as a function of waiting time for an $Fe_3Al$ reference sample as well as the studied MG under three different conditions.

# Supplementary Note 3

The main manuscript displays MD simulation results for a shear strain rate of $10^7\ s^{-1}$ in Figure 7. Here we show that a two orders of magnitude slower strain rates yields qualitatively the same result. Supplementary Figure 3 shows the same panel as in Figure 7, but for a rate of $10^5\ s^{-1}$, where again fluctuations along the stress-strain curve are well aligned with fluctuations of the $\varepsilon_{0.8}$ relaxation strain. Due to the low shear strain rate, the stress-strain response does not exhibit a strong initial stress overshoot as seen in Figure 7a $(10^7\ s^{-1})$ and shows evidence of multiple serration activity similar to the well-known serrated response known for strain localization of MGs[5].

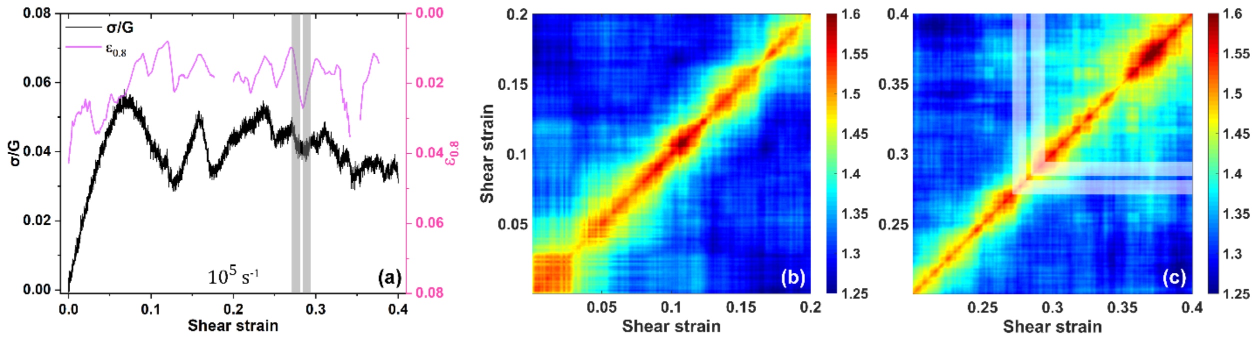


*Supplementary Figure 3:* ***Atomistic simulations reveal origin of intermittent dynamics*** *(a) Normalized shear stress along with* $\varepsilon_{0.8}$ *momentary relaxation strains as a function of shear strain for a binary model glass under simple shear. Note the inverted axis on the relaxation strain. (b) A Two-Strain Correlation-Function from 0 to 0.2 in shear strain shows the transition from elastic to plastic flow at a shear strain of ca. 0.035, followed by fluctuations in relaxation strain with plastic flow. (c) Shear strains from 0.2 to 0.4 show fluctuating relaxation strains, decorrelation-recorrelation, as well as variation in contrast along the main diagonal.*

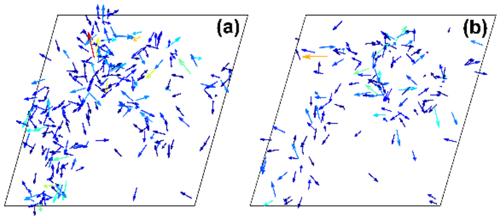


*Supplementary Figure 4:* ***Localization of microplastic activity*** *Displacement maps comparing the atomic displacements caused by a 0.01 increment in strain at a strain rate of* $10^5\ s^{-1}$*. The strain ranges are indicated in Supplementary Figure 3a and 3c. (a) and (b) exemplify fast and slow dynamics, respectively. The corresponding strain regions are highlighted in the stress-strain graphs in Figure S4.*

It should be noted that the apparent stress drops in Supplementary Figure 3a are not due to the experimentally seen thermally-activated shear-band instabilities[6], but rather a signature of varying intrinsic resistance to collective shear-driven disordering. The much lower strain rate furthermore allows a larger amount of intrinsic relaxation during the shear deformation, which naturally leads to less spatially-confined collective atomic motion. Despite this, the lower strain rate reinforces the conclusion made in the main manuscript: the pinching of the TSCF reflects collective plastic activity with larger mean displacements, whereas a broadening is seen when structural events are more dilute and of smaller displacement magnitude. This is again highlighted in Supplementary Figure 4, which displays the displacement maps for the strain intervals indicated in Supplementary Figure 3a and 3c. A narrow segment of the TSCF corresponds to extended plastic activity with larger displacements and is linked to a stress drop in the stress-strain response. Broadening of the TSCF is related to dilute plastic activity and smaller displacement magnitudes.

## Supplementary Methods

The presently used Lennard-Jones potential,

$$V_{ab}(r) = 4\varepsilon\left(\left(\frac{\sigma_{ab}}{r}\right)^{12} - \left(\frac{\sigma_{ab}}{r}\right)^{6}\right) \quad \text{(Supplementary Equation 2)}$$

describes a model binary atomic system. We use the two-atom-type Wahnström parameterizaton[4], in which $r$ is the distance between atoms of type a and b (equal to either type 1 or type 2). $\varepsilon$ sets the energy scale, $\sigma_{11}$ sets the length scale, and the masses of the atom types are set to $m_1 = 2m_2$. When converted to metal specific systems, 1 billion MD steps corresponds to approximately one micro-second of physical simulation time.

The two presented shear deformation simulations, at the strain rates $10^7\ s^{-1}$ and $10^5\ s^{-1}$, (see Figure 7 and Supplementary Figure 3 ) both show an initial linear elastic response, followed by a peak stress region and a subsequent flow stress region. The initial peak stress region is common to all atomistic simulations of deformation below the glass transition temperature and is characteristic of the high strain rates used.

As discussed in the methods section, simulated speckle patterns at a time $t$ are calculated via

$$|S(q,t)|^2 = \left|\sum_{i=1}^{N} f_i e^{i\vec{q}\cdot\overrightarrow{r_i(t)}}\right|^2 \quad \text{(Supplementary Equation 3)}$$

where $\vec{q} = \begin{bmatrix} q_{n_x} & q_{n_y} & 0 \end{bmatrix}$, and $\overrightarrow{r_i(t)}$ is the position of the $i^{th}$ atom at time $t$. This $xy$ plane corresponds to the plane of the applied shear stress. Because of the finite size of our periodic cell (defined by periodicity lengths $L_x$, $L_y$ and $L_z$), $q_{n_x} = \frac{2\pi}{L_x n_x}$ and $q_{n_y} = \frac{2\pi}{L_y n_y}$ where $n_x$ and $n_y$ are integers. Thus, the scattering vector has units of inverse $\sigma_{11}$. Since we consider a model system which is therefore not material specific, we set the atomic form factor to unity for the present work. Supplementary Figure 5a shows a typical speckle pattern calculated prior to loading showing the main structural ring. Supplementary Figure 5b displays a similar speckle pattern at a strain corresponding to the plastic flow region. For this latter speckle pattern, the affine displacement due to the elastic load has been removed to avoid ring distortion. This is done since the present work focuses only on how plastic atomic-scale processes (whatever their origin) affect the XCPS signal. In Supplementary Figure 5, only the pixels that enter into the evaluation of the two-strain correlation function (TSCF) are shown.

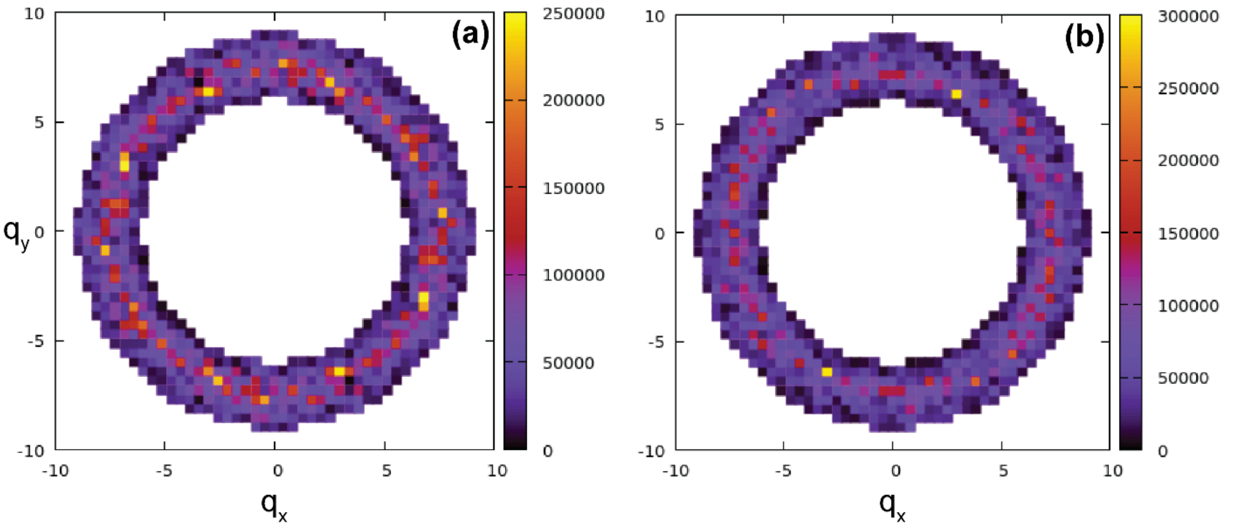


*Supplementary Figure 5:* **Speckle patterns from atomistic simulations** Speckle pattern corresponding to the full ring of the first peak of $S(q,t)$ is shown for two different times and thus shear strains. The color scale indicates the intensity.

The TSCF is a pixel-wise comparison of speckle patterns as shown in Supplementary Figure 5, from two different strains. Given the constant strain rate in the MD simulations, a TSCF is equivalent to a two-time correlation function (TTCF), after a linear rescaling of the axes. This allows for direct comparison with the experimental TTCFs.

## Supplementary References

1. Madsen A, Leheny RL, Guo H, Sprung M, Czakkel O. Beyond simple exponential correlation functions and equilibrium dynamics in x-ray photon correlation spectroscopy. *New J Phys* **12**, 055001 (2010).

2. Küchemann S, Liu C, Dufresne EM, Shin J, Maaß R. Shear banding leads to accelerated aging dynamics in a metallic glass. *Phys Rev B* **97**, 014204 (2018).

3. Derlet PM, Maaß R. Thermally-activated stress relaxation in a model amorphous solid and the formation of a system-spanning shear event. *Acta Mater* **143**, 205-213 (2018).

4. Wahnström G. Molecular-dynamics study of a supercooled two-component Lennard-Jones system. *Phys Rev A* **44**, 3752-3764 (1991).

5. Maaß R, Löffler JF. Shear-Band Dynamics in Metallic Glasses. *Adv Funct Mater* **25**, 2353-2368 (2015).

6. Maaß R, Klaumünzer D, Löffler JF. Propagation dynamics of individual shear bands during inhomogeneous flow in a Zr-based bulk metallic glass. *Acta Mater* **59**, 3205-3213 (2011).
